# Exposure to the Florida red tide dinoflagellate, *Karenia brevis*, and its associated brevetoxins induces ecophysiological and proteomic alterations in *Porites astreoides*

**DOI:** 10.1371/journal.pone.0228414

**Published:** 2020-02-07

**Authors:** David A. Reynolds, Mi-Jeong Yoo, Danielle L. Dixson, Cliff Ross

**Affiliations:** 1 Department of Biology, University of North Florida, Jacksonville, Florida, United States of America; 2 Department of Biology, Clarkson University, Potsdam, New York, United States of America; 3 School of Marine Science and Policy, University of Delaware, Lewes, Delaware, United States of America; Living Oceans Foundation, TAIWAN

## Abstract

As reef-building corals are increasingly being exposed to persistent threats that operate on both regional and global scales, there is a pressing need to better understand the complex processes that diminish coral populations. This study investigated the impacts of the Florida red tide dinoflagellate *Karenia brevis* and associated brevetoxins on selected facets of coral biology using *Porites astreoides* as a model system. When provided with choice assays, *P*. *astreoides* larvae were shown to actively avoid seawater containing red tide (5×10^5^ cells L^-1^–7.6×10^6^ cells L^-1^) or purified brevetoxins (0.018 μg mL^-1^ brevetoxin-2 and 0.0018 μg mL^-1^ brevetoxin-3). However, forced exposure to similar treatments induced time-dependent physiological and behavioral changes that were captured by PAM fluorometry and settlement and survival assays, respectively. Adult fragments of *P*. *astreoides* exposed to red tide or associated brevetoxins displayed signs of proteomic alterations that were characterized by the use of an iTRAQ-based quantitative proteomic analysis. The novel use of this technique with *P*. *astreoides* demonstrated that protein regulation was highly contingent upon biological versus chemical treatment (*i*.*e*. live *K*. *brevis* vs. solely brevetoxin exposure) and that several broad pathways associated with cell stress were affected including redox homeostasis, protein folding, energy metabolism and reactive oxygen species production. The results herein provide new insight into the ecology, behavior and sublethal stress of reef-building corals in response to *K*. *brevis* exposure and underscore the importance of recognizing the potential of red tide to act as a regional stressor to these important foundation species.

## Introduction

The prevalence of toxic red tide events in the state of Florida, stemming from the dinoflagellate *Karenia brevis*, has received considerable attention over the past several decades [1–3]. While large and persistent blooms of *K*. *brevis* are known to occur on an annual basis on Florida’s Gulf Coast, data suggest that red tides are increasing both spatially and temporally likely as a function of enhanced nutrient availability through increased urbanization and agricultural expansion [1, 4]. Blooms are initiated offshore yet are typically transported by physical means onto the West Florida Shelf inshore environment. In addition, *K*. *brevis* cells can be advected into the Florida Keys via the combined factors of prevailing winds and proximity to the Gulf of Mexico (GOM) Loop Current [5, 6]. Both of these regions represent complex mosaics of coral reefs and epibenthic communities that have the capacity to be adversely affected by high concentrations of *K*. *brevis* and their associated brevetoxins [7, 8].

Brevetoxins are a group of neurotoxic, lipid soluble cyclic polyethers with their nomenclature (PbTx-1, -2, -3, etc.) derived from *Ptychodiscus brevis*, a prior epithet for *K*. *brevis*. These compounds function as sodium channel depolarizing agents that show high affinity towards site 5 of the alpha-subunit of voltage-gated sodium channels (VGSC) [9,10]. Through their depolarizing capabilities, brevetoxins represent one of the most common chemical stressors affecting the Florida coastline by promoting fish kills, marine mammal mortality events and adversely affecting public human health [11, 12]. While as many as 12 different analogs have been reported to exist through cellular metabolism and environmental turnover [12, 13], brevetoxin-2 (PbTx-2) and brevetoxin-3 (PbTx-3) are considered to be the most abundant species detected in water and air samples during blooms [14–17].

A substantial amount of research has been carried out on the deleterious effects of *K*. *brevis* and associated brevetoxins on epibenthic macroinvertebrates, in particular commercially relevant bivalve mollusks [18–21]. However, there continues to be a considerable gap in our understanding of how red tides impact selected facets of basic coral biology. This is quite striking given that the Fish and Wildlife Research Institute (FWRI) and the National Oceanic and Atmospheric Administration’s Harmful Algal Blooms Observing System (NOAA HABSOS) frequently report *K*. *brevis* in proximity to coral-dense areas of the GOM and, to a lesser extent, the Florida Keys (http://myfwc.com/redtidestatus) [8, 22]. Furthermore, as coral reefs undergo rapid degradation due to the negative effects of ocean acidification and elevated sea surface temperatures [23, 24], these same global scale stressors have the capability of profoundly impacting *K*. *brevis* growth and toxicity [25, 26]. Stochastic disturbances such as *K*. *brevis* blooms may not only have a negative impact on the health of adult reef-building corals but can also affect early critical life history stages that drive recruitment. Thus, understanding how red tide exposure affects varying life-history stages of corals represents a critical piece of information required to predict the fate of Florida’s coral reefs. We have previously demonstrated that short term exposure of *Porites astreoides* larvae to naturally occurring concentrations of *K*. *brevis* results in enhanced oxidative stress and a depression in respiration [27]. These findings prompted this current study, which further explored the effects of live *K*. *brevis* and purified brevetoxin analogs on selected ecophysiological, behavioral and proteomic endpoints in *P*. *astreoides*.

Identifying and quantifying the degree of sub-lethal stress in corals can be accomplished by using a variety of techniques including biochemical and physiological approaches [28–30]. However, with the technological advancements in high-throughput sequencing and analysis of large-scale biological datasets, there has been an increase in the use of -omics technologies to assess the cellular effects of environmental stressors on coral species. The annotation and quantitation of transcriptomes through Next Generation Sequencing technology (NGS) has been a common strategy for measuring gene expression in selected coral species [31–36]. This is because NGS enables a *de novo* transcriptome assembly on non-model organisms without a reference genome and can also be used to examine the diversity of alternatively spliced transcripts [37–41]. This technique has been used to advance our understanding of numerous cellular processes in corals, such as the molecular components of coral-symbiont interactions, gene activation in coral bleaching and biomarkers of certain stressors [34–36]. While transcriptomics has proven to be a useful tool in the study of coral gene expression, there are limitations. For example, relative abundance of transcripts can only be regarded as an estimate of the cellular response since many factors can influence the final proteins produced, such as post-transcriptional regulation, post-translational modifications and protein degradation [42]. Because of this, proteomics allows for a more realistic “picture” of the functional cellular response following a selected treatment.

Although many studies have used transcriptomic techniques in corals and anemones, few studies have utilized the proteome to identify cellular responses to stress [43–46]. This is unfortunate given the low congruency between mRNA expression and protein concentration in corals and, thus, the true phenotypic status may not be captured [43, 44]. The overall lack of proteomic-based studies may be attributed to the fact that most species are non-model organisms and proteomics requires a reference database of theoretic protein spectra in order to establish protein identification. In addition, the robustness of proteins recovered is reliant upon the inclusiveness of genes represented in the reference database. A workaround to this issue can be accomplished by integrating RNA-sequencing techniques to generate a sample-specific protein database, a technique termed Proteomics Informed by Transcriptomics (PIT) [47]. This approach can be more efficient than using genome data alone in three main ways: 1) it can incorporate and identify nonsynonymous single nucleotide polymorphisms, which would otherwise alter peptide properties of mass and / or charge; 2) it can incorporate mRNA isoforms that are absent from the reference genome; and 3) it can estimate transcript abundance to identify which transcripts are expressed, which can be used to trim search databases and reduce the likelihood of type 1 errors from multiple peptide comparisons [48–51]. The use of RNA-sequence derived databases in proteomics has also shown to be effective in protein identification in the absence of a genome reference [52, 53]. With the popular use of transcriptomic studies on coral species throughout the literature, there is a large amount of assembled transcriptome data publicly available. Constructing a protein search database through published RNA-sequence data can provide a unique opportunity for protein identification in coral proteomics analysis.

Given the paucity of data available on the effects of red tide on corals, we undertook this current study to gain a better understanding of how *K*. *brevis* and associated brevetoxins impact coral biology using *Porites astreoides* as a model species. *P*. *astreoides* was chosen because this species is commonly found in tropical and subtropical areas and has been well-studied with respect to the end-points assessed in this work including larval metamorphosis and settlement, mortality and response to the presence of macroalgae and cyanobacteria [54–56]. Using a multi-pronged approach, including quantitative proteomic analysis, our results provide new insight into the ecology, behavior and sublethal stress of corals in response to red tide exposure.

## Materials and methods

### *Karenia brevis* culture maintenance and brevetoxins

Cultures of *K*. *brevis*, strain CCFWC257, were acquired from the Florida Fish and Wildlife Research Institute and maintained at room temperature under full-spectrum lighting (100–120 μmol m^-2^s^-1^) on a 12:12 h light:dark photoperiod. Cultures were grown in GP media consisting of seawater (salinity of 35; made with Instant Ocean Sea Salt, Instant Ocean ®) amended with soil extract obtained from garden soil [57]. Purified PbTx-2 and -3 were purchased from MARBIONC Development Group LLC (Wilmington, NC, USA). Brevetoxin solutions were made by first suspending PbTx-2 and PbTx-3 analogs in 100% methanol to a concentration of 0.1 μg μL^-1^ and 0.01 μg μL^-1^, respectively. Subsequently, solutions were diluted in seawater to a final concentration of 0.018 μg mL^-1^ PbTx-2 and 0.0018 μg mL^-1^ PbTx-3. These values represent ecologically relevant concentrations that have been previously quantified in water samples collected from *K*. *brevis* blooms off the coast of Florida [58]. To account for potential impacts of methanol on coral behavior and physiology, a methanol control was used in the following experiments at a concentration of 0.0004% v/v.

### Collection of *Porites astreoides* coral and larvae

Colonies of *P*. *astreoides* were collected at a 6-m depth from Wonderland Reef (24°733.62’ N, 81°730.08’ W) in the lower Florida Keys, transported to Mote’s Elizabeth Moore International Center for Coral Reef Research & Restoration (Summerland Key, FL) in coolers and kept in raceway tables with running seawater. Permission for collection (permit no. FKNMS-2016-023) was provided by the Florida Keys National Marine Sanctuary. All colonies were maintained at a constant salinity of 35 and were shaded allowing penetration of 10% photosynthetic active radiation (< 200 μmol m^-2^s^-1^). Photosynthetic photon flux fluence rates (PPFFR) were measured using a LI-193 underwater spherical quantum sensor in conjunction with a LI-250A light meter (LI-COR, Lincoln, NE, USA). Coral larvae were collected between the nights of May 23 and May 25, 2017, during the new moon (May 25), when adult colonies release brooded larvae [59]. To obtain larvae, each adult colony was placed in an individual 3-liter Rubbermaid Grip n’ Mix bowl supplied with continuously running seawater. The bowls were tilted so the positively buoyant larvae spilled over the handles of the bowls each night into plastic tri-pour beakers, which in turn were fitted with 180-μm mesh bottoms. The water level inside the tank was maintained at 15 cm, so the larvae remained in the tri-pour beakers until sunrise the next morning. Following collection, larvae were pooled to randomize possible maternal effects. One-day-old larvae (newly released) were always used for any given experiment. In addition, five adult colonies were chosen at random for exposure treatments and cut into 3 cm^2^-fragments with a circular saw. Fragments were allowed to acclimate for 24 h in raceway tables prior to use in any experiment. One fragment from each colony was randomly assigned to a treatment and exposure time (experiments described below). Following larval release, colonies were returned to the site of collection and reattached to the benthos with Z-Spar Splash Zone Compound underwater epoxy (U.S. Spars, Gainesville, FL).

### Larvae behavior

In order to test the ability of *P*. *astreoides* larvae to discriminate between red tide contaminated seawater and uncontaminated seawater, planula were placed in a two-channel Atema choice flume using protocols previously described [60]. For each trial, artificial seawater (Instant Ocean^®^; salinity of 32) contained either varying concentrations of live *K*. *brevis* that represented high (7.6x10^6^ cell L^-1^, 3.8x10^6^ cell L^-1^, 2.5x10^6^ cell L^-1^) or medium (5x10^5^ cell L^-1^) bloom densities or a fixed concentration of brevetoxins (0.018 μg mL^-1^ PbTx-2 combined with 0.0018 μg ml L^-1^ PbTx-3) and was tested in parallel with untreated artificial seawater or artificial seawater treated with methanol (brevetoxin trials: 0.0004% v/v). Water flow was measured using a flow meter and maintained at 100 mL min^-1^ per channel for each trial. Dye tests were used to ensure laminar flow between the two channels, without turbulence or eddies. Preliminary tests were performed to ensure larvae were able to maintain position and make forward progress against the flow before any trials were conducted. For each trial a minimum of 10 random larvae were selected. Only larvae able to maintain their position in the flume were recorded. Individual larvae were pipetted into the center of the flume where they were free to swim towards a preferred water source. Specimens were allowed to acclimate for 1 min, followed by a 2-min testing period where the position (left or right) of the larvae was recorded at 5-s intervals. To ensure there was no side bias being observed larvae were given a 1-minute rest period, during this time water was flushed and the sources switched. The larvae were pipetted back into the flume for an additional 1-minute acclimation period followed by a 2-minute testing period.

### *Karenia brevis* and brevetoxin exposure assays

In order to assess the maximum quantum efficiency of photosystem (PS) II in symbionts from larval and adult corals (details described below) as well as changes in the adult coral proteome following exposure to *K*. *brevis* and associated toxins, a two-factorial design was implemented using time and treatment as fixed factors. Treatments included: ambient seawater, methanol control (0.0004% v/v), purified brevetoxins (0.018 μg mL^-1^ PbTx-2 combined with 0.0018 μg mL^-1^ PbTx-3) and *K*. *brevis* (2.5x10^6^ cells L^-1^). Each replicate (n = 5/treatment), containing either 115 larvae or a 3 cm^2^-fragment, was placed in a 400-mL tri-pour beaker at a final volume of 300 mL. Forty 7-L plastic aquaria were placed in two raceway flow tables (used as water baths to maintain consistent temperatures) and filled with seawater. In turn, each aquarium served as a housing unit to hold four tri-pour beakers. Each beaker was randomly assigned a treatment and an aquarium and was held in place by the use of wooden clothes pins. Temperatures of the aquaria were continuously monitored using a YSI model 85 multiprobe meter (YSI, Yellow Springs, OH, USA). Larvae and adult fragments were allowed to incubate for 24 or 48 hours. Post-exposure, 15 larvae were used for the analysis of maximum quantum efficiency of PSII photochemistry, while the remaining 100 larvae were flash frozen in liquid nitrogen (N_2_) and stored at -80°C for future analysis. Adult fragments were analyzed for changes in maximum quantum efficiency of PSII photochemistry and then flash frozen in liquid N_2_ and stored at -80°C for proteomic analysis.

### Photochemical efficiency

Following exposure, coral fragment replicates and larvae were placed in 300 mL and 1 mL seawater, respectively. Photochemical efficiency was evaluated using pulse amplitude modulated (PAM) fluorometry (Diving PAM; Walz, Germany). Corals were dark adapted for one hour prior to analysis and changes in maximal quantum yield (F_v_/F_m_ = (F_m_-F_o_)/F_m_) of photosystem II (PSII) were measured to indicate disruption of photochemical performance. For larvae, readings were taken by pipetting 15 larvae in 25 μl of seawater onto the tip of the fiber optic cable. The PAM fluorometer’s gain and intensity were both set to 7. Following dark-adaptation, adult fragments were submerged in seawater in a 3 L Rubbermaid Grip’s Mix bowl ® with the PAM fluorometer’s gain and intensity set to 3.

### Larvae survival and settlement kinetics

To test the effects of *K*. *brevis* or brevetoxin exposure on planula survival and settlement, larvae were exposed to the same treatments as described above. In these assays, larvae (50 larvae per replicate) were placed in a 400 mL tri-pour beaker containing a single terracotta tile (4.5 cm x 4.5 cm x 1 cm; Sunshine Pavers®), which served as a settlement substrate. Prior to use, all tiles were preconditioned at a depth of 6 m offshore (24°733.62’ N, 81°730.08’ W) for 4 weeks. Beakers were filled to a final volume of 300 mL. Larvae were exposed to their respective treatments for 24, 48 and 72 hours (n = 5/treatment). Following exposure, the number of total surviving larvae (swimmers + settlers) were divided by 50 (the initial number of larvae) to determine the percentage survival, and the number of larvae that had settled and metamorphosed were divided by 50 to determine the percentage settlement.

### Protein extraction

Coral fragments that were incubated for 48 h were subjected to proteomic analysis. Previously frozen samples were thawed and tissue was removed from the calcium carbonate skeleton over ice using a Paansche airbrush (Paansche, Inc., Chicago, IL, USA) and homogenate buffer (50 mM phosphate buffer, (pH 7.8) with 0.05 mM dithiothreitol). Four biological replicates were used per treatment (total = 20). Total protein was isolated from crude tissue homogenates (~2 g of coral tissue) as described previously [61] with the following modifications. Tissue was ground in liquid N_2_ with a pre-cooled mortar and pestle in 6 mL of extraction buffer (1.2% β-mercaptoethanol, 0.1 M Tris-HCl (pH 8.8), 10 mM EDTA, 0.9 M sucrose) and 6 mL Tris-saturated phenol (pH 8.8), followed by overnight incubation at room temperature with shaking. All chemicals were obtained from Millipore Sigma (St. Louis, MO, USA) unless noted otherwise. Samples were centrifuged at room temperature for 40 min at 5,000 g. The resulting protein pellets were dissolved in 4 M urea and 0.1% SDS in 10 mM Tris-HCl (pH 8.0). Protein concentration was measured using the EZQ Protein Quantification Kit (Thermo Fisher Scientific, Inc., San Jose, CA, USA) with SoftMax Pro Software v5.3 (Molecular Devices, Inc., San Jose, CA, USA).

### iTRAQ labelling and proteomic analysis

For each adult coral sample, 100 μg of protein was reduced, alkylated to block cysteine residues, digested with 10 μg of sequencing grade trypsin (1:10 ratio; Promega, Madison, WI, USA) and labelled using the iTRAQ® Reagents 8plex kit as per the manufacturer’s instructions (Sciex, Inc., Foster City, CA, USA). In each set, two independent samples of ambient seawater controls were labeled with iTRAQ tags 113 and 117, two independent samples of methanol control were labeled with iTRAQ tags 114 and 118, two independent brevetoxin treatments were labeled with iTRAQ tags 115 and 119 and two independent *K*. *brevis* treatments were labelled with iTRAQ tags 116 and 121. The combined peptide mixtures were lyophilized and a solid phase extraction was performed in order to remove impurities by use of a SOLA SPE cartridge (Thermo Fisher Scientific) according to the manufacturer’s instructions. The resulting purified peptide mixtures were dissolved in strong cation exchange (SCX) solvent A (25% (v/v) acetonitrile, 10 mM ammonium formate, and 0.1% (v/v) formic acid pH 2.8). The peptides were eluted with a linear gradient of 0–20% solvent B (25% (v/v) acetonitrile and 500 mM ammonium formate at pH 6.8) over 50 min followed by a ramp up to 100% solvent B over the course of 5 min and held for an additional 10 min using an Agilent HPLC system 1260 outfitted with a polysulfoethyl A column (2.1 mm × 100 mm, 5 μm, 300 Å, PolyLC, Columbia, MD, USA). The absorbance at 280 nm and 214 nm were monitored, and a total of 14 fractions were collected.

The fractions were lyophilized and resuspended in LC solvent A (0.1% formic acid in 97% water (v/v), 3% acetonitrile (v/v)). A hybrid quadrupole Orbitrap (Q Exactive) MS system (Thermo Fisher Scientific) was used with high energy collision dissociation (HCD) in each MS and MS/MS cycle. The MS system was interfaced with an automated Easy-nLC 1000 system (Thermo Fisher Scientific). Each sample fraction was loaded onto an Acclaim Pepmap 100 pre-column (20 mm × 75 μm; 3 μm-C18) and separated on a PepMap RSLC analytical column (250 mm × 75 μm; 2 μm-C18) at a flow rate at 350 μl min^-1^ using a linear gradient of solvent A (0.1% formic acid (v/v)) to 30% solvent B (0.1% formic acid (v/v) and 99.9% acetonitrile (v/v)) for 95 min, to 98% solvent B for 15 min, and hold 98% solvent B for an additional 30 min. Full MS scans were acquired in the Orbitrap mass analyzer over m/z 400–2000 range with resolution 70,000 at 200 m/z. The top ten most intense peaks with charge state ≥ 3 were fragmented in the HCD collision cell normalized collision energy of 28%, (the isolation window was 2 m/z) as previously described [62]. The maximum ion injection times for the survey scan and the MS/MS scans were 250 ms, respectively and the ion target values were set to 3 x 10^6^ and 1 x 10^6^, respectively. Selected sequenced ions were dynamically excluded for 60 sec.

### Protein database construction, identification and analysis

The raw MS/MS data files were processed by a thorough database search approach, considering biological modification and amino acid substitution, against a nonredundant consensus database (six-framed 575,500 contigs; uploaded in a data depository, see below). The database was constructed from three previously published and annotated transcriptomes of holobiont *Porites* species [36, 63–64]. As a symbiont transcriptome was not explicitly queried in this study, the results provided herein chiefly reflect alterations in the host cnidarian proteome. Peptide MS/MS data were searched against the database with Fraglet and Taglet searches under the Paragon algorithm using ProteinPilot version 4.5 software (AB Sciex, Inc.) [65]. The following parameters were considered for all searches: fixed modification of methylmethane thiosulfonate-labeled cysteine, fixed iTRAQ modification of amine groups in the N-terminus lysine and variable iTRAQ modifications of tyrosine. The raw peptides were identified with at least six amino acids under the Paragon algorithm. The ProteinPilot cut-off score (unused) was set to 1.3, which corresponds to a confidence level of 95% and a 5% false discovery rate (FDR) [66].Relative quantification of proteins detected by unique peptides was conducted using ratios from tandem mass spectra. After confirming that there were no variations in ratios using the ambient controls (113, 117) as denominators, the ratios of the other six samples were calculated against the average of the two controls. For a protein to be considered significantly differentially concentrated, it must have been quantified with at least three peptides in biological tetraplicates, with a Fisher’s combined probability of < 0.05 and a fold change > 1.5 or < 0.5. For protein quantification, only MS/MS spectra that were unique to a particular protein and where the sum of the signal-to-noise ratios for all the peak pairs > 9 were used for quantification. Sequences of identified proteins were annotated using Blast2GO suite (http://www.blast2go.com/b2ghome) [67]. Where applicable, differentially concentrated proteins were assigned classes as per the Gene Ontology (GO) Consortium [68]. MS proteomics data were deposited in the ProteomeXchange Consortium [69] via the MassIVE partner repository with the data set identifiers PXD015741 and MSV000084432.

### Statistical analysis

For Atema choice flume experiments Kolmogorov-Smirnov tests were used to compare the proportion of time that individuals spent in the stream of water containing the olfactory cue (*K*. *brevis* or brevetoxins) compared to the proportion of time that individuals spent in one side of the chamber when no cue was present (seawater vs. seawater). Maximum quantum yield of PSII of *P*. *astreoides* larvae was analyzed using a two-way ANOVA with time and treatment as fixed factors, followed by Tukey’s post hoc test. For adult samples, the data failed the assumption of normality and a Sheirer-Ray-Hare nonparametric two-way ANOVA was used [70]. To further discern what factors were contributing to differences among groups, a Mann-Whitney U test among treatment levels was conducted with Bonferroni corrected p-values to reduce the effects of family-wise error of multiple hypothesis testing. Data for larval settlers and survivors were arcsine square root transformed since they were percentages. For settlers, a two-way ANOVA was used followed by Tukey’s post hoc test. For survivors, the data failed the assumption of normality and a Sheirer-Ray-Hare nonparametric two-way ANOVA was used and a Mann-Whitney U test was used to compare means among treatment levels with a Bonferroni corrected p-value.

## Results

### Larvae behavior

**Fig 1:**
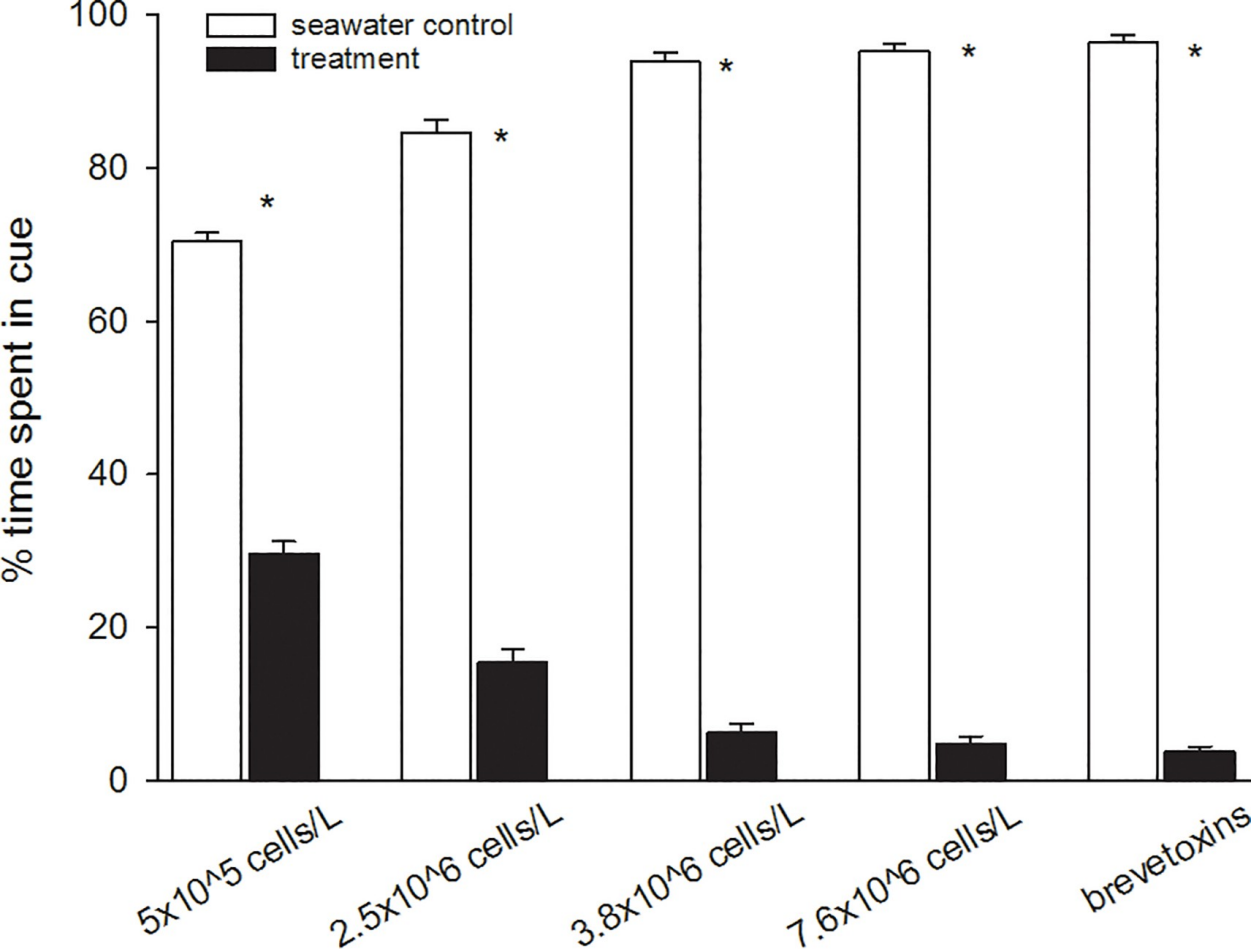
Percent time *Porites astreoides* larvae spent in a channel when introduced to a pairwise choice between two water sources of ambient seawater and a biological cue. Trials consisted of seawater containing either *Karenia brevis* cells that represented medium (5 × 10^5^ cells L^-1^) and high (2.5 × 10^6^ cells L^-1^, 3.8 × 10^6^ cells L^-1^ and 7.6 × 10^6^ cells L^-1^) bloom densities; or brevetoxins at 0.018 μg mL^-1^ PbTx-2 and 0.0018 μg mL^-1^ PbTx-3. The brevetoxin seawater control contained methanol at a concentration of 0.0004% v/v. “*” indicates p < 0.001 via Kolmogorov Smirnov test when compared to control trials of ambient seawater vs. ambient seawater. Bars represent + 1 SE.

The proportion of time that individual larvae spent in a channel containing either *K*. *brevis* or brevetoxins was compared to the proportion of time that individuals spent in one side of the chamber when no cue was present (seawater vs. seawater). The mean proportion of time spent in one side of the chamber was near 50% (mean = 49.31%, standard error (SE) = + 1.33) for the control trial of seawater vs. seawater. Larvae showed strong preference for untreated seawater in each trial, with a larger percent time spent in the ambient seawater with increasing concentration of *K*. *brevis* (Fig 1). At the highest concentration of *K*. *brevis* (7.6 × 10^6^ cells L^-1^), larvae displayed the most discernable avoidance with a mean time in the contaminated cue of 4.80% (SE = + 1.03, p < 0.0001). Larvae exposed to 3.8 × 10^6^ cells L^-1^, 2.5 × 10^6^ cells L^-1^ and 5 × 10^5^ cells L^-1^ spent 6.25% (SE = + 1.16), 15.42% (SE = + 1.75) and 29.58% (SE = + 1.67) of the time in those cues, respectively (p < 0.0001 per trial). Larvae exposed to the brevetoxin mixture (0.018 μg mL^-1^ PbTx-2 and 0.0018 μg PbTx-3 mL^-1^) spent 3.75% of the time in that cue compared to the methanol control (SE = + 0.75, p < 0.0001).

### Photochemical efficiency

**Fig 2:**
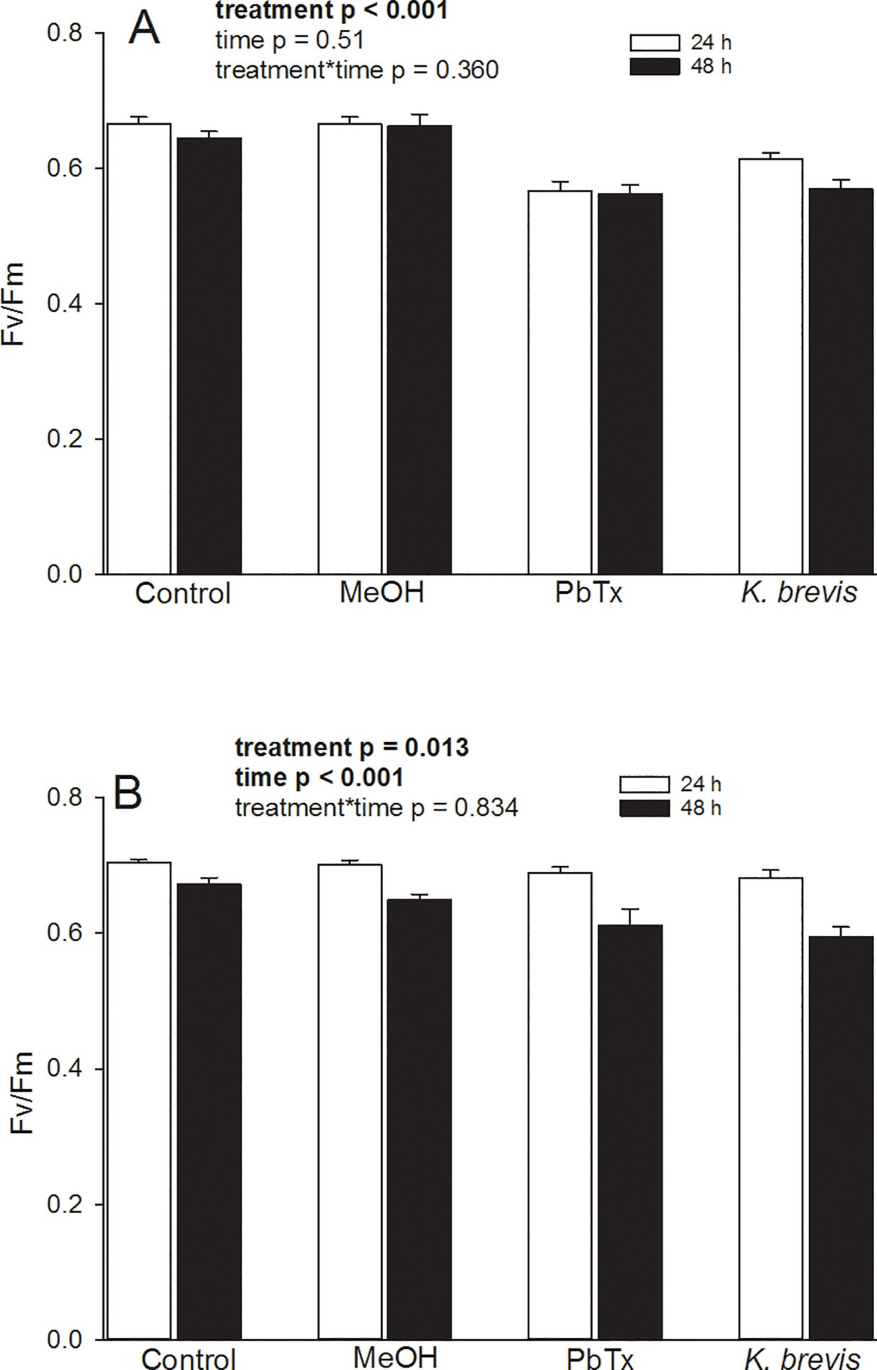
Maximum quantum yield of *in hospite* Symbiodiniaceae within *Porites astreoides* tissue following exposure to *Karenia brevis* or brevetoxins: (A) *Porites astreoides* larvae, (B) *Porites astreoides* adults. MeOH = methanol, PbTx = brevetoxin. Bars represent + 1 SE.

Maximum quantum yield (F_v_/F_m_) was used to determine if *K*. *brevis* or associated brevetoxins had a negative impact on the PSII photochemistry of *in hospite* Symbiodiniaceae. In larvae, there were significant differences in fluorescent yield as a function of treatment (F _(3,32)_ = 27.531, p < 0.001), yet not time (F _(1,32)_ = 4.110, p = 0.51) (Fig 2A). Furthermore, there was no significant interaction between factors (F _(3,32)_ = 1.108, p = 0.360). It was noted that larvae exposed to *K*. *brevis* or brevetoxins had a reduced maximum quantum yield compared to seawater or seawater-methanol controls. The photochemical efficiency of adult *P*. *astreoides* was significantly affected by treatment (H = 7.887, p = 0.048) and time (H = 20.916, p < 0.001) but there was no significant interaction (treatment*time) (H = 0.676, p = 0.879) (Fig 2B). Further nonparametric analyses among groups following a Bonferroni correction showed that there was a reduction in the maximum quantum yield of PSII in the *K*. *brevis* treatment when compared to the control group. A summary of post hoc analyses of pairwise comparisons of maximum quantum yield data is shown in S1A and S1B Table.

### Larvae survival and settlement kinetics

**Fig 3:**
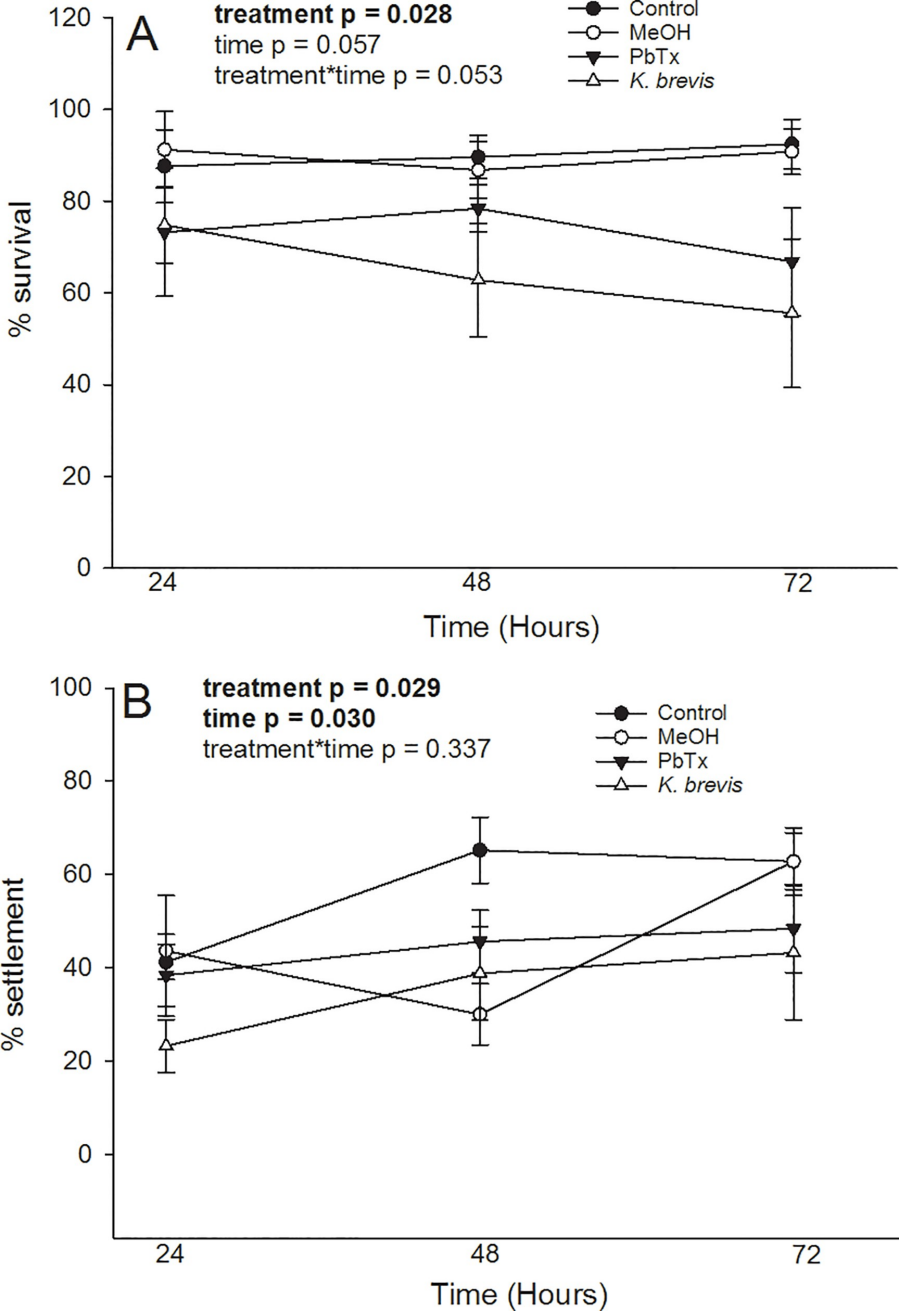
Kinetics of larval settlement and survival following exposure to either ambient seawater, methanol (MeOH), brevetoxins (PbTx) or *Karenia brevis*; for either 24, 48 and 72-hours: (A) percent survival, and (B) percent settlement. Bars represent ± 1 SE.

The proportion of individuals surviving (Fig 3A) and settling (Fig 3B) was significantly impacted as a function of treatment (H = 9.079, p = 0.028; and F _(3,48)_ = 3.264, p = 0.029, respectively). While time had a significant effect on the number of larvae that settled (F _(2,48)_ = 3.782, p = 0.030), it only had a marginal effect on survival (H = 5.729, p = 0.057). There was no interaction among treatments (survival: H = 12.42, p = 0.053; and settlement: F _(6,48)_ = 1.172, p = 0.337). By use of a Bonferroni post hoc test it was determined that both settlement and survival were significantly reduced following exposure to *K*. *brevis* compared to ambient seawater controls (S1C and S1D Table). At any time point, the survivorship of methanol-treated larvae (MeOH controls) failed to differ from larvae exposed to seawater controls. By the end of the experiment (72 h), survival levels of individuals that were exposed to *K*. *brevis* were 37% lower than survival levels of individuals in controls maintained in ambient seawater.

### Protein database construction and protein profiling

Three available transcriptomes of *Porites* species were translated using six-frame translation and constructed into one protein search database (*Porites* species concatenated nonredundant; S2 Table). Search results based on the four databases showed that the constructed database provided the highest number of hits, which identified a total of 1,371 proteins with a 5% FDR. Out of the proteins identified, 1,290 proteins were quantified and further statistically analyzed. BLAST searches against the NCBI nonredundant protein sequence (nr_v5) database resulted in 1,335 BLAST hits, with 957 proteins annotated and 213 proteins mapped (S1 Fig). The majority of sequences showed homology to sequences associated with other corals, providing evidence of the database’s integrity (S1 Fig).

### Comparative proteomics of exposure to *Karenia brevis* and brevetoxin analogs

**Fig 4:**
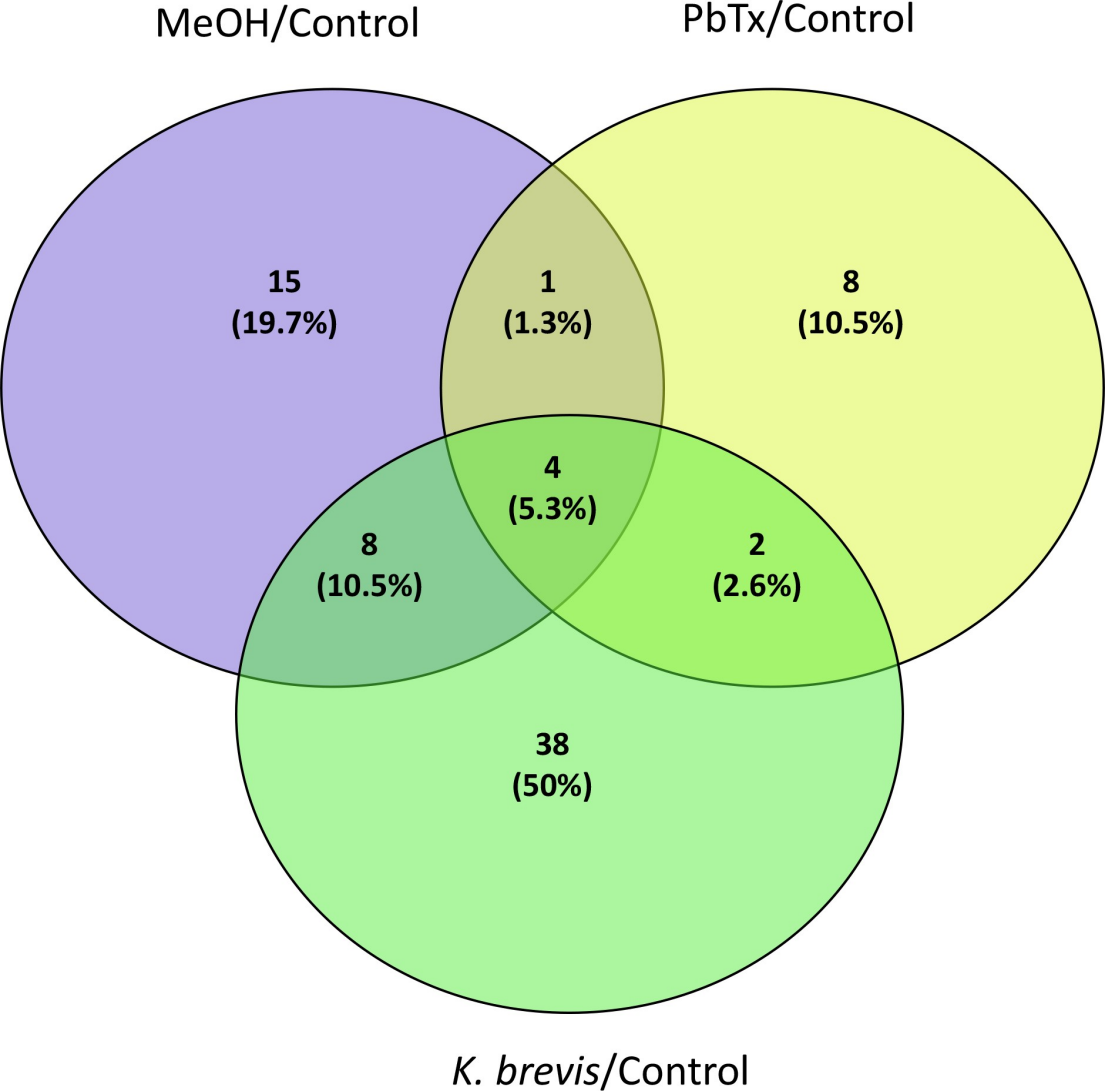
Venn diagram of differentially concentrated proteins among treatments. A total of 52, 15 and 28 proteins were significantly up- or downregulated in *Karenia brevis*, brevetoxin (PbTx) and methanol (MeOH) treatments, respectively.

**Fig 5:**
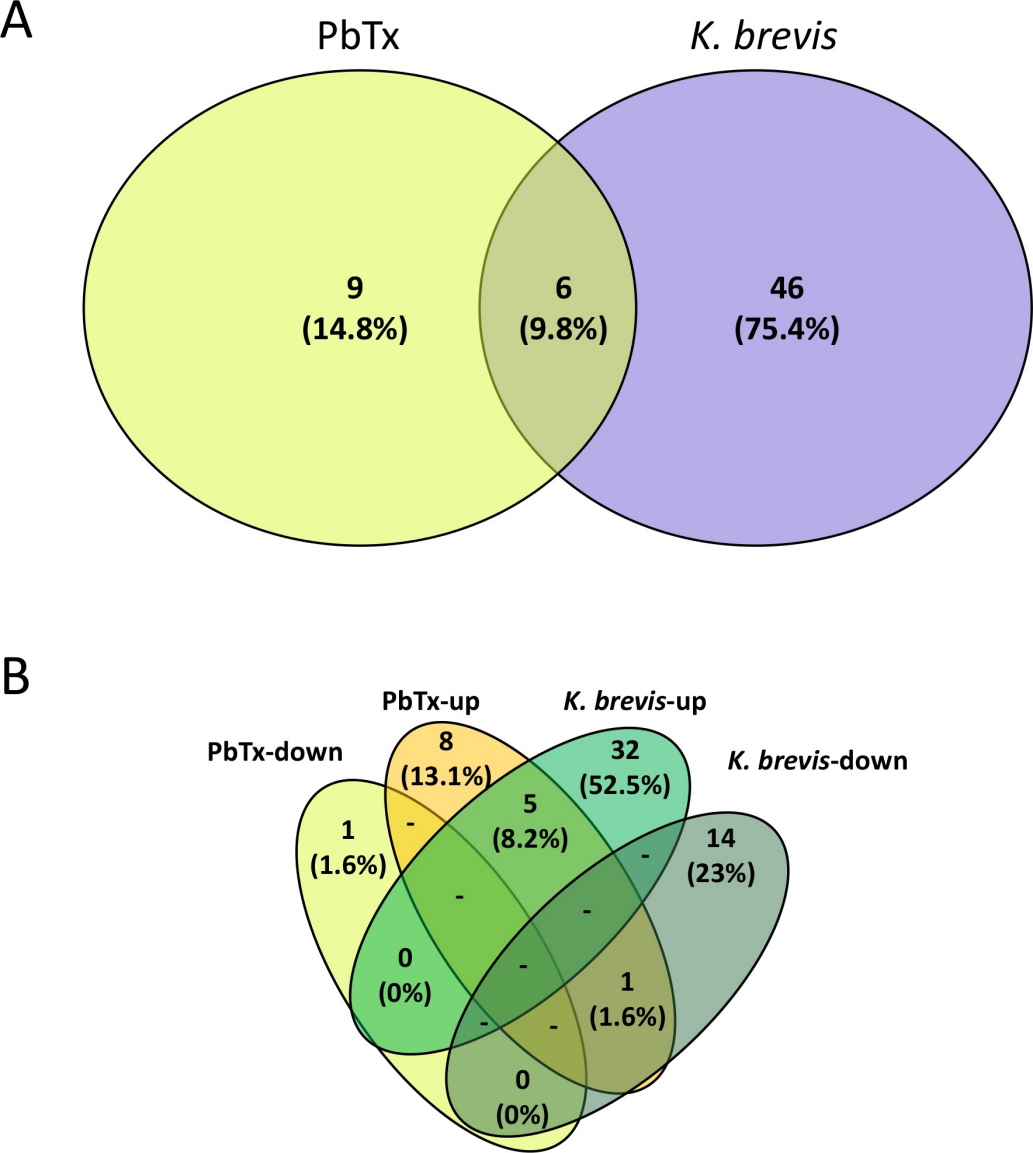
Venn diagram showing the number of differentially concentrated proteins in *Porites astreoides* tissue following exposure to red tide and associated brevetoxins: (A) total number of differentially concentrated proteins in brevetoxin (PbTx) or *Karenia brevis* treatments; and (B) proteins grouped into up- or downregulated categories in response to brevetoxin or *Karenia brevis* treatments.

The iTRAQ labelling and the LC-MS/MS methods allowed for the simultaneous protein quantitation in treatments (MeOH, brevetoxin, and *K*. *brevis*) compared to controls in order to determine the differential expression of proteins following exposure. Exposure to methanol (methanol control) resulted in the differential concentration of 28 proteins compared to controls. However, only 5 of these proteins overlapped with samples exposed to the brevetoxin treatment. Queries failed to find annotated functions for 3 out of the 5 proteins. The two overlapping proteins with functional assignments were involved in calcium binding (cluster 201619) and lipid transport (cluster 30), respectively (S3 Table). The brevetoxin and *K*. *brevis* treatments showed 15 and 52 proteins that were differentially concentrated, respectively (Fig 4; S3 Table). The *K*. *brevis* treatment contained 15 downregulated and 37 upregulated proteins, while the brevetoxin treatment had one downregulated and 14 upregulated proteins, compared to the control (Table 1). There were six differentially concentrated proteins shared between the two treatments (Fig 5; Table 1). Out of the 61 differentially concentrated proteins found in both the brevetoxin and *K*. *brevis* treatments, 32 and 14 proteins were uniquely upregulated (*e*.*g*., disulfide isomerases, NAD(P) transhydrogenase, cathepsins, and myosin-2 essential light chain) and downregulated (*e*.*g*., 3-phosphoinositide-dependent protein kinase 1), respectively, in the *K*. *brevis* treatment. In comparison, the brevetoxin treatment had eight and one proteins that were uniquely upregulated (*e*.*g*., histone H2A-like, Histone H2B, and Histone H4-like), and down regulated (cluster 599840), respectively. Within the six differentially concentrated proteins shared between the two treatments, five proteins (cluster 30, cluster 78396, cluster 153015, cluster 97665 and cluster 78285) with function in skeletal organic matrix, notch signaling and axon development, were upregulated within both treatments and one protein (cluster 45626) was upregulated in the brevetoxin treatment and downregulated in the *K*. *brevis* treatment with functions in actin assembly, microtubule motility and protein complexes for gene expression. While 178 out of the identified 1,371 proteins (13%) showed strong homology to Symbiodiniaceae, only 3 of these were differentially regulated in response to brevetoxin or live *K*. *brevis* treatment (Table 1).

**Table 1:** List and annotation of 61 differentially concentrated proteins of the coral species *Porites astreoides* following exposure to brevetoxins (PbTx) or *Karenia brevis* cells. Proteins in colored cells indicate either upregulated (red) or downregulated (blue) compared to control samples. FC represents fold change in treated samples relative to control samples. Bold indicates sequences from Symbiodiniaceae. Listed p-values are associated with Fisher’s exact test.

Gene ID	Accession #	Sequence Description	PbTx		*K*. *brevis*	
			FC	p-value	FC	p-value
Cluster599840	---Not Available---	---Not Available---	0.18	0.0322	1.64	0.2997
Cluster45626	gi|1176095254	actin-related protein 2	1.56	0.0219	0.40	0.0131
Cluster30	gi|156221418	predicted protein	1.57	0.0308	11.32	0.0041
Cluster78396	gi|1176105383	uncharacterized skeletal organic matrix protein 5-like	11.00	0.0494	9.72	0.0467
Cluster153015	gi|1191069530	uncharacterized protein LOC110251054	5.23	0.0000	3.31	0.0000
Cluster97665	gi|1176107158	failed axon connections homolog	4.64	0.0112	3.02	0.0378
Cluster78285	gi|1176094913	neurogenic locus Notch protein-like	1.63	0.0074	2.53	0.0000
Cluster74461	gi|1005492381	histone H2A-like	1.71	0.0242	1.56	0.5163
**Cluster72128**	**gi|1129189947**	**photosystem I subunit II (chloroplast)**	2.12	0.0161	1.34	0.2022
Cluster120960	gi|156384837	histone H4-like	2.02	0.0000	1.30	0.4262
Cluster54901	gi|134104063	chloroplast soluble peridinin-chlorophyll a-binding protein precursor	1.74	0.0000	1.29	0.0016
Cluster92319	gi|156308410	histone H2B	3.02	0.0128	1.12	0.1788
Cluster42011	gi|1176106978	trichohyalin-like isoform X2	1.72	0.0282	1.08	0.9878
Cluster201619	gi|1005436313	Calreticulin	1.61	0.0215	0.80	0.0633
Cluster480843	---Not Available---	---Not Available---	1.85	0.0274	0.59	0.0190
Cluster12861	gi|1176065290	vacuolar protein sorting-associated protein 35	1.05	0.0226	0.49	0.0000
Cluster86218	gi|1005455133	centromere-associated protein E-like	1.06	0.2853	0.48	0.0207
Cluster77817	gi|1176083360	thiosulfate sulfurtransferase-like	1.30	0.2002	0.47	0.0483
Cluster70872	gi|1176102334	6-phosphogluconate dehydrogenase, decarboxylating-like	1.31	0.1303	0.45	0.0492
Cluster44622	gi|1176099874	protein kinase C and casein kinase substrate in neurons protein 1 isoform X3	1.22	0.2016	0.44	0.0437
Cluster36510	gi|1176074793	3-phosphoinositide-dependent protein kinase 1	1.29	0.2687	0.43	0.0460
Cluster14994	gi|1176081911	spermatogenesis-associated protein 20	0.96	0.3760	0.42	0.0433
Cluster38861	gi|514686106	hypothetical protein PTSG_08380	1.38	0.2078	0.41	0.0404
Cluster39017	gi|1263131715	Trifunctional enzyme subunit beta, mitochondrial	0.93	0.1247	0.40	0.0029
Cluster90375	gi|1005435074	proteasomal ubiquitin receptor ADRM1-like	0.72	0.1822	0.40	0.0396
Cluster72289	gi|1005478902	prohibitin-2 isoform X1	1.37	0.1582	0.38	0.0403
Cluster13855	gi|1176083171	centrosomal protein of 290 kDa-like	0.79	0.0213	0.36	0.0082
**Cluster21163**	**gi|452944860**	**photosystem I P700 apoprotein A2 (chloroplast)**	1.22	0.1645	0.33	0.0359
Cluster77998	gi|1176061994	Krueppel-like factor 16	1.14	0.2920	0.32	0.0214
Cluster135790	gi|1005469133	Seed maturation protein domain protein	0.87	0.0882	5.46	0.0194
Cluster563359	gi|999976565	succinate--CoA ligase (ADP/GDP-forming) subunit alpha, mitochondrial-like	0.92	0.5393	2.99	0.0236
Cluster12612	gi|156224519	Endoplasmin	1.25	0.2646	2.73	0.0000
Cluster6698	gi|999985476	NAD(P) transhydrogenase, mitochondrial	1.13	0.7138	2.69	0.0019
Cluster8701	gi|1176081387	cleavage stimulation factor subunit 2-like	1.06	0.3771	2.65	0.0355
Cluster110390	gi|931444489	metal-dependent hydrolase	1.41	0.0079	2.56	0.0058
Cluster665	---Not Available---	---Not Available---	1.05	0.3370	2.55	0.0155
**Cluster57194**	**gi|1129196481**	**2-hydroxyacid dehydrogenase**	0.71	0.2976	2.49	0.0479
Cluster3401	gi|828223890	THO complex subunit 2	0.87	0.3766	2.46	0.0322
Cluster71042	gi|156376666	heterogeneous nuclear ribonucleoprotein A/B isoform X1	0.98	0.4789	2.46	0.0406
Cluster137452	gi|1005485438	myosin-2 essential light chain-like	1.19	0.1023	2.43	0.0008
Cluster68537	gi|1176073868	cartilage matrix protein-like	1.21	0.1569	2.43	0.0000
Cluster1877	gi|1176099008	Intersectin-1	1.37	0.1259	2.33	0.0111
Cluster60925	gi|1263126539	Cathepsin B	1.32	0.3480	2.28	0.0013
Cluster89694	gi|1270045038	Proteasome subunit alpha type-5	1.11	0.8611	2.18	0.0125
Cluster63543	gi|1176121623	cathepsin L1-like	1.00	0.7961	2.18	0.0346
Cluster80745	gi|1176123940	cytochrome b-c1 complex subunit Rieske, mitochondrial-like	1.17	0.8346	1.94	0.0001
Cluster24085	gi|944358134	Tubulin alpha-1C chain	0.83	0.8934	1.91	0.0291
Cluster29482	gi|1270036269	glucosidase 2 subunit beta-like	0.91	0.3125	1.91	0.0448
Cluster10800	gi|156219690	coatomer subunit gamma-2	0.66	0.0663	1.90	0.0005
Cluster98716	gi|1176090182	ras-related protein Rab-11A	1.01	0.6742	1.90	0.0098
Cluster40025	gi|1263121217	Protein disulfide-isomerase A6	1.07	0.5999	1.82	0.0031
Cluster101211	gi|1176117806	glutathione S-transferase-like	0.83	0.6735	1.73	0.0023
Cluster136968	gi|1176092520	neuronal pentraxin-2-like	1.26	0.1302	1.72	0.0289
Cluster111088	gi|1005447951	NADH dehydrogenase (ubiquinone) 1 beta subcomplex subunit 8, mitochondrial	1.61	0.1944	1.71	0.0095
Cluster58031	gi|1263138886	protein disulfide-isomerase tigA precursor	0.97	0.8207	1.70	0.0065
Cluster41678	gi|1005486638	phosphatidylserine decarboxylase	1.00	0.7788	1.68	0.0018
Cluster5009	gi|1176107568	dyp-type peroxidase family protein	1.03	0.0076	1.64	0.0010
Cluster15920	gi|1176120735	predicted protein	1.08	1.0000	1.57	0.0133
Cluster32856	gi|1176110238	protein disulfide isomerase	1.22	0.2482	1.56	0.0060
Cluster225948	gi|1101333444	fructose-bisphosphate aldolase	0.87	0.0222	1.52	0.0000
Cluster20910	gi|749717734	Transposon Tf2-6 polyprotein	0.94	0.0713	1.52	0.0002

### Functional analysis of differentially concentrated proteins

**Fig 6:**
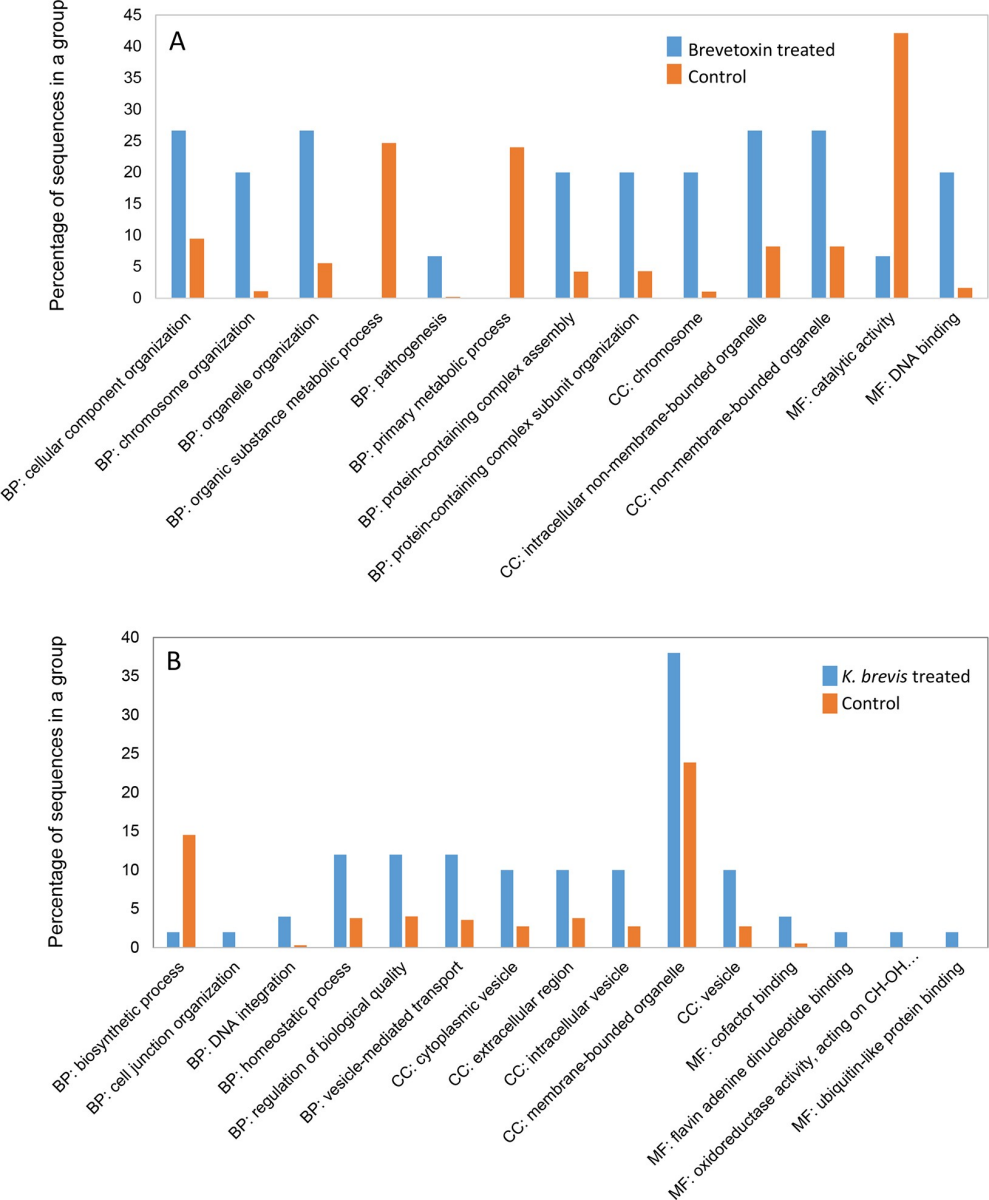
Enrichment bar charts of differentially concentrated proteins in *Porites astreoides* as a function of treatment: (A) 15 differentially concentrated proteins in brevetoxin-treated samples relative to controls, (B) 52 differentially concentrated proteins in *Karenia brevis*-treated samples compared to controls. BP, CC and MF represent biological process, cellular component and molecular function, respectively.

Cellular responses varied depending on whether corals were exposed to brevetoxins or *K*. *brevis*. Differentially concentrated proteins were categorized into one of three GO groupings as described below. Coral tissue exposed to brevetoxins showed an increase in concentration of proteins associated with biological processes (chromosome organization, protein-containing complex assembly, pathogenesis, organelle organization, cellular component organization and protein-containing complex subunit organization), cellular compartment (chromosomes, non-membrane-bound organelles and intracellular non-membrane-bound organelles) and molecular function (DNA binding) (Fig 6A). In contrast, corals exposed to brevetoxins underwent a downregulation in proteins associated with catalytic activity (molecular function) primary metabolic processes and organic substance metabolic process (the latter two pertaining to biological processes). Within the *K*. *brevis* treatment, upregulated proteins were associated with biological processes (DNA integration, vesicle-mediated transport, regulation of biological quality, cell junction organization and homeostatic processes), cellular compartment (cytoplasmic vesicles, membrane-bounded organelles, extracellular region, vesicles and intracellular vesicles) and molecular function (ubiquitin-like protein binding, cofactor binding, flavin adenine dinucleotide binding and oxidoreductase activity) (Fig 6B). Downregulation was detected in biosynthetic processes.

## Discussion

### Larvae behavior

*Porites astreoides* larvae actively avoided water contaminated with either *K*. *brevis* cells or brevetoxins. This effect was observed even at the lowest concentration assessed (5 × 10^5^ cells l^-1^), which is characterized as “medium bloom density” by the Florida Fish and Wildlife Conservation Commission (FWC). The concentrations used in this study fall within the range of blooms that have been previously reported within 15 km of the shoreline (in regions where corals are likely to inhabit) [1]. The larvae also demonstrated a clear tendency of avoiding seawater contaminated with brevetoxins with concentrations, 0.018 μg mL^-1^ PbTx-2 and 0.0018 μg mL^-1^ PbTx-3, lower than what is typically detected under medium bloom density conditions.

Active avoidance of red tide blooms by planktonic organisms have been previously reported in larval vertebrates and arthropods [71, 72]. Given that coral larvae can respond in a similar manner suggests that key elements of this behavior may have arisen early on in animal phylogenies and may have broader evolutionary implications relating chemical cues to habitat site selection. Coral larvae have been shown to be chemically attracted to seawater obtained from healthy coral-dominated field sites, while exposure to seawater from degraded reef locations (typically dominated by benthic algae) has a strong deterrent effect [60]. These types of observations, coupled with recent genomic advances have allowed for a better understanding of the cnidarian nervous system structure and function [73, 74]. It is becoming clear that coral planula have a high degree of nervous system complexity, which enables them to modify their behavior in response to selected environmental cues.

### Photochemical efficiency

Chemical extracts derived from diverse assemblages of marine macroalgae have been shown to repress photochemical activity in corals [75]. However, to our knowledge, this is the first report documenting the effects of naturally-occurring brevetoxins and *K*. *brevis* on coral photochemistry. The maximum quantum yield of PSII of both larvae and adults were significantly affected by both types of treatments. Previous work has demonstrated that high cell counts of *K*. *brevis* (4 x 10^6^ cells L^-1^) or brevetoxin concentrations (ranging between 5–15 μg L^-1^) can significantly increase lipid hydroperoxide content in adult specimens of *P*. *astreoides* [27]. While the negative correlation between oxidative stress and maximum quantum yield of PSII is particularly intriguing, it is not known if brevetoxins directly affect photosystem electron transport or if there are indirect effects (*i*.*e*. bleaching or compromised membrane integrity) that impact photochemical efficiency. The mechanism(s) of inhibition most certainly warrant further investigation.

### Larvae survival and settlement kinetics

Along with dispersal, successful recruitment is contingent upon the larvae’s ability to locate a suitable substrate and complete metamorphosis. In the current study, it was shown that exposure to high bloom concentrations of *K*. *brevis* impact coral larvae survival and settlement, while not necessarily impacting the rate of settlement. For example, if larvae exhibited a postponement of settlement in response to this stressor, it would be expected that there would be a higher number of swimmers in the brevetoxin and *K*. *brevis* treatments. Alternatively, if larvae used settlement and metamorphosis as a way to escape being suspended in contaminated surface waters, it would be expected that there would be fewer swimmers present. The percentage of swimmers was relatively constant throughout each treatment, this suggests that the presence of *K*. *brevis* did not postpone or accelerate settlement. Although there was a higher number of swimmers in the ambient seawater control when compared to the *K*. *brevis* treatment at 72 hours, this was due to *K*. *brevis*-induced mortality. For both brevetoxin and *K*. *brevis* treatments, there was a reduction in percent settlement and survivorship at 48 and 72 hours, compared to ambient seawater controls. Settlement remained constant between the 48 and 72-hour exposures for ambient seawater and *K*. *brevis* treatments, suggesting settlement occurred early within the study. However, a decline in survivorship was noted as a function of time for larvae treated with brevetoxin and *K*. *brevis* while survivorship levels of larvae under ambient seawater and methanol conditions remained relatively constant. These trends became even more apparent when strictly comparing larvae exposed to ambient seawater versus *K*. *brevis*. Since there was a similar decline in swimmers for each treatment, it can be inferred that the rate of attempted settlement was similar regardless of presence of a stressor. However, the fact that there was a reduction in both settlement and survivorship in the presence of a stressor suggests that *K*. *brevis* causes larval mortality shortly before or after settlement.

Settlement and metamorphosis are energetically costly events and during this transition larvae may be vulnerable to the impacts of surrounding stressors [76]. Before settlement, the larvae may be able to sustain metabolic activities that allow them to tolerate cellular stress. However, when metabolic activities switch to promote settlement, the reallocation of energy may result in a particularly susceptible period for the larvae. Regardless of the underlying cause, this study demonstrated that exposure to *K*. *brevis* can have negative impacts on larval survival. In a previous study it was found that a 20-hour exposure to low (6 × 10^5^ cells L^-1^) and high (4 × 10^6^ cells L^-1^) concentrations of *K*. *brevis* or cell lysates (prior to being introduced to a settlement chamber) did not impact *P*. *astreoides* larvae settlement or survival [27]. In this study, the 24-hour exposure had very little difference in percent mortality and percent settlement for all four treatments, while at 48 hours there was a discernible difference. At acute exposures, larvae may be resistant to the negative effects of *K*. *brevis*, while prolonged exposure results in a negative impact on recruitment success. This possibility warrants further investigation since red tide blooms can last up to 18 months [5].

### Proteomics

One of the goals of this study was to test the feasibility of using previously published transcriptomes of *Porites* spp. to construct a reference search database for protein identification in the broad-scale proteomic analysis of *P*. *astreoides*. Using a protein database constructed of three *Porites* transcriptomes accompanied by iTRAQ labelling techniques, a total of 1,371 proteins were identified with 1,290 proteins quantifiable. While 61 differentially concentrated proteins were identified in response to brevetoxin or *K*. *brevis* exposure, these findings may actually serve as an underestimate for two main reasons. First, the database used in this study was derived from transcriptomic information which represents select portions of the genome transcribed under specific conditions. Thus, our searches only targeted a portion of the entire proteome. Second, in some cases iTRAQ technology has been reported to underestimate the actual fold-change in abundance of some proteins compared to label-free proteomic techniques [77–81]. Despite these challenges, our findings demonstrate that the use of transcriptomes coupled with iTRAQ technology can still serve as a valuable tool to assess coral protein regulation in response to environmental stressors, particularly red tide exposure.

While this study focused upon the proteomic alterations encountered in the cnidarian host, the coral holobiont represents a dynamic system consisting of the coral animal as well as the associated microorganisms including bacteria, archaea, fungi, viruses and dinoflagellate algae. Alterations in protein abundance, stemming from any of the aforementioned partners can drive holobiont homeostasis [82]. Select stressors, such as elevated temperature, have been shown to cause notable shifts in the Symbiodiniaceae proteome when compared to the host coral [43]. In this current study, only 3 out of 61 differentially concentrated proteins were found to be linked to *Symbiodinium* in response to brevetoxin or live *K*. *brevis* treatments. It is very likely that if Symbiodinaceae transcriptomes were specifically queried, many more *Symbiodinium* proteins would have been identified. In Florida, the majority of shallow and mid-depth colonies of *P*. *astreoides* have been noted to contain *Symbiodinium* A4 or A4a [83]. This clade is thought to be one of the more robust clades associated with bleaching resistance and enhanced photoprotection [84]. Brevetoxin treatment caused an increase in photosystem I subunit II protein, while *K*. *brevis* treatment caused a decrease in photosystem I P700 apoprotein A2 protein and a concomitant increase in 2-hydroxyacid dehydrogenase. Collectively, this suggests that light harvesting and at least some redox homeostatic activities were being modulated by the algal symbiont. In order to provide a more thorough assessment of the effects of red tide and associated brevetoxins on Symbiodinaceae protein responses, future work is most certainly warranted.

The differential abundance of *P*. *astreoides*’ proteins was dependent upon whether the coral was being exposed to whole *K*. *brevis* cells or purified brevetoxin analogs. Exposure to live *K*. *brevis* mainly caused the differential abundance of proteins related to biosynthesis (downregulation), vesicle-mediated transport (upregulation) and homeostatic processes (upregulation). Conversely, brevetoxin treatment caused a notable upregulation in proteins associated with DNA binding and DNA organization. Interestingly, only six of the identified proteins overlapped between the brevetoxin and *K*. *brevis* treatments, while nine and forty six proteins were uniquely differentially concentrated in brevetoxin and *K*. *brevis* treatments, respectively. The differential expression of proteins in adult *P*. *astreoides* following *K*. *brevis* exposure suggests that several broad pathways associated with cell stress were being affected including redox homeostasis, protein folding, energy metabolism and reactive oxygen species (ROS) production (Table 1). Many cellular processes rely upon the maintenance of intracellular redox potential and changes may result in cellular dysfunction or apoptosis. Pathways of protein synthesis located in the endoplasmic reticulum (ER) are particularly sensitive to redox fluctuations [85–86]. There were a number of proteins associated with redox homeostasis and ER dysfunction that were upregulated in response to *K*. *brevis* exposure including disulfide isomerases and endoplasmin (Table 1). Disulfide isomerases are involved in the formation and breakage of disulfide bonds during protein synthesis. This family of proteins has been shown to be induced and modified during oxidative stress, which is likely a response to ensure proper protein folding during oxidation of the ER [87–88]. In addition, expression of endoplasmin has been shown to suppress oxidative damage and stabilize calcium homeostasis in the ER following stress [89–90].

Along with the disruption of redox homeostasis, proteins associated with energy metabolism were affected when *P*. *astreoides* was exposed to live *K*. *brevis*. The induction of energy metabolism during the cellular stress response may be necessary for producing reducing equivalents (*e*.*g*., NADH, NADPH) which are required to fuel antioxidant systems. Energy expenditures may also increase in response to protein degradation, protein chaperoning and DNA repair [91]. Furthermore, ER stress and activation of unfolded protein response pathways (UPR; responsible for directing misfolded proteins towards degradation and not secretion) have been linked to the expression of genes in glucose metabolism [92–93]. In this current study, many proteins involved in energy metabolism were upregulated following exposure to *K*. *brevis*, including those that possess key roles in glycolysis, the citric acid cycle, and electron transport chains within mitochondria (NADH dehydrogenase (ubiquinone) 1 beta subcomplex subunit 8, NAD(P) transhydrogenase, succinate-CoA ligase (ADP/GDP-forming) subunit alpha, cytochrome b-c1 complex subunit Rieske and fructose-bisphosphate aldolase) (Table 1). Many of these proteins are susceptible to oxidative damage or have known roles in ROS defense [94–101]. For example, NADH dehydrogenase (ubiquinone) 1 beta subcomplex subunit 8 is associated with the NADH dehydrogenase complex 1, which is the first enzyme in the mitochondrial electron transport chain. This enzyme is particularly susceptible to oxidative damage, which results in enzyme inactivation [95]. Other enzymes can further exacerbate ROS release in the mitochondria following oxidative damage, such as cytochrome b-c1 complex subunit Rieske, which is a component of the mitochondrial ubiquinol-cytochrome c reductase complex dimer (complex III dimer) and is involved in the generation of the electrochemical potential in the mitochondria for ATP synthesis. Not only is this protein found to be a target of oxidative damage but its dysfunction is also implicated in the release of ROS in the mitochondria [98, 101]. Other identified proteins were noted to have roles in response to oxidative stress, acting as antioxidants or alternative pathways of energy production, such as NAD(P) transhydrogenase, fructose-bisphosphate aldolase, glutathione S-transferase predicted protein and dyp-type peroxidase family protein [94, 96, 99, 100, 102, 103]

Proteins involved in protease activity were also upregulated following exposure to *K*. *brevis*. The upregulation of cathepsin B and cathepsin L1 suggests the induction of protein degradation, which can provide further evidence of ER and mitochondrial dysfunction, as well as oxidative stress. In addition to functioning in the UPR response in the ER, proteases actively recognize and degrade oxidized proteins, which can be re-synthesized *de novo* [104–109]. Thus, the increased presence of proteases detected may be an artifact of ER and mitochondrial breakdown.

The disruption of intra- and intercellular Ca^2+^ maintenance may serve as the basis for the broad proteomic responses detected in *P*. *astreoides* following *K*. *brevis* exposure. ER stress and oxidative damage can result in the release of Ca^2+^ from the ER into the cytosol. This can cause enhanced production of mitochondrial-based ROS which, in turn, can result in a feedback loop, promoting further Ca^2+^ release from the ER [108–115]. In this study there is indirect support for increased cytosolic Ca^2+^ movement following exposure to *K*. *brevis* as evidenced by the downregulation of 3-phosphoinositide-dependent protein kinase 1 (which modulates the release of sequestered calcium ions into the cytosol) and upregulation of myosin-2 essential light chain (which functions in calcium binding). This falls in line with previous work reporting that gill and brain tissue of fish exposed to PbTx-1 display proteomic alterations in the expression of calcium binding proteins (Myosin-like proteins) [116]. Based on the overall cellular functions observed in this study, *K*. *brevis* may be impacting cytosolic Ca^2+^ levels, resulting in subsequent ER/mitochondrial dysfunction, increased oxidative stress and protein damage. Such changes in Ca^2+^ can directly impact ER oxidative homeostasis, resulting in misfolded proteins, induction of UPR, as well as increased production of ROS within the mitochondria.

Interestingly, patterns of proteomic changes were not shared between *K*. *brevis* and brevetoxin treatments. The majority of proteins differentially concentrated from exposure to the brevetoxin analogs PbTx-2 and PbTx-3 were related to DNA organization, chromatin formation and transcription expression (*e*.*g*. histone H4, histone H2A, histone H2B) (Table 1). Histone variants play an important role in differential gene expression. Furthermore, histone modification can alter nucleosome conformation and change the accessibility of selected transcriptional regulatory proteins [117–121]. Deregulation and overexpression of certain histones have been reported to be associated with cancerous cells and have been known to cause abnormal oncogene expression, which can be suppressed by degradation of the histone through upregulation of tumor suppressors [122–126]. In this study, the brevetoxin-induced upregulation of histones could be attributed to cellular proliferation as has been reported in other studies. For example, it has been found that exposure to low concentrations of PbTx-2 (10^−8^ M) results in the proliferation of Jurkat E6-1 cells, yet at high concentrations cell division is negatively impacted [127]. This type of hormetic response is thought to be an adaptive compensatory response of the cell; however, the mechanisms that regulate this are still poorly understood [128]. Future work integrating a model system such as *Aiptasia* sp. would likely offer additional insight into the proteomic responses of cnidarians when exposed to red tide.

In conclusion, the results of this study provide evidence that ecologically relevant concentrations of *K*. *brevis* and the most abundant analogs of brevetoxins (PbTx-2 and -3) have negative impacts on the recruitment, viability and sub-lethal stress response of *P*. *astreoides*. As reef-building corals are increasingly exposed to persistent threats that operate on both regional and global scales there is a pressing need to better understand the complex processes that diminish coral populations. Depending upon the duration of exposure and the intensity of bloom conditions, red tide events have the capacity to be hazardous to both pelagic and benthic life history stages of corals.

## Supporting information

S1 Table Results of post hoc analysis (p-values) of pairwise comparisons between means of treatments (control, MeOH control, brevetoxin and *K*. *brevis*) following two-way ANOVA (A,C) or Sheirer-Ray-Hare nonparametric two-way ANOVA (B,D): A) Tukey’s HSD post hoc pairwise comparisons among treatments of photochemical efficiency in *P*. *astreoides* larvae; B) Mann-Whitney U post hoc pairwise comparisons among treatments of photochemical efficiency in *P*. *astreoides* adults with Bonferroni corrected p-values; C) Tukey’s HSD post hoc pairwise comparisons among treatments of larval settlement in *P*. *astreoides*; D) Mann-Whitney U post hoc pairwise comparisons among treatments of larval survival in *P*. *astreoides* with Bonferroni corrected p-values. Asterisk (*) represents a two-tailed significance at α = 0.05. Redundant comparisons are shaded in gray.
(DOCX)

S2 Table Database information for publicly available transcriptomes of *Porites* species and the resulting constructed database. (DOCX)

S3 Table List and annotation of 76 differentially concentrated proteins of the *P. astreoides* holobiont following exposure to MeOH, brevetoxin or *K. brevis* cells. Highlighted proteins indicate either significant up- (red) or down-regulation (blue). Proteins associated with Symbiodiniaceae are highlighted in yellow.
(XLSX)

S1 Fig Distribution of Blast2GO search results of the 1,371 proteins identified. (TIF)

S2 Fig Distribution of species from BLAST Top-hits. A total of 1,371 protein sequences were identified using the constructed database and were blasted against the NCBI nonredundant protein sequence (nr_v5) database. The majority of hits showed homology to corals including *Orbicella faveolata* and *Acropora* sp.
(TIF)
