# Supplementary material for: Exposure to the Florida red tide dinoflagellate, Karenia brevis, and its associated brevetoxins induces ecophysiological and proteomic alterations in Porites astreoides
Source: PLoS One. 2020 Feb 7;15(2):e0228414. doi: 10.1371/journal.pone.0228414 (PMC7006924; doi:10.1371/journal.pone.0228414)
Supplement: S1 Table — Results of post hoc analysis (p-values) of pairwise comparisons between means of treatments (control, MeOH control, brevetoxin and K. brevis) following two-way ANOVA (A,C) or Sheirer-Ray-Hare nonparametric two-way ANOVA (B,D): A) Tukey’s HSD post hoc pairwise comparisons among treatments of photochemical efficiency in P. astreoides larvae; B) Mann-Whitney U post hoc pairwise comparisons among treatments of photochemical efficiency in P. astreoides adults with Bonferroni corrected p-values; C) Tukey’s HSD post hoc pairwise comparisons among treatments of larval settlement in P. astreoides; D) Mann-Whitney U post hoc pairwise comparisons among treatments of larval survival in P. astreoides with Bonferroni corrected p-values. Asterisk (*) represents a two-tailed significance at α = 0.05. Redundant comparisons are shaded in gray. (DOCX) [file pone.0228414.s001.docx]

Supplemental Table 1. Results of post hoc analysis (p-values) of pairwise comparisons between means of treatments (control, MeOH control, brevetoxin and *K. brevis*) following Two-Way ANOVA (A,C) or Sheirer-Ray-Hare nonparametric Two-Way ANOVA (B,D): A) Tukey’s HSD post hoc pairwise comparisons among treatments of photochemical efficiency in *P. astreoides* larvae; B) Mann-Whitney U post hoc pairwise comparisons among treatments of photochemical efficiency in *P. astreoides* adults with Bonferroni corrected p-values; C) Tukey’s HSD posthoc pairwise comparisons among treatments of larval settlement in *P. astreoides* D) Mann-Whitney U post hoc pairwise comparisons among treatments of larval survival in *P. astreoides* with Bonferroni corrected p-values. Asterisk (*) represents a two-tailed significance at α = 0.05. Redundant comparisons are shaded in gray.

| Larvae PAM | Ambient Seawater | MeOH | Brevetoxin | *K. brevis* |
| --- | --- | --- | --- | --- |
| **Ambient** | - | 0.902 | *<0.001 | *<0.001 |
| **MeOH** | - | - | *<0.001 | *0.001 |
| **Brevetoxin** | - | - | - | 0.188 |
| **K. brevis** | - | - | - | - |

A)

B)

| Adult PAM | Ambient Seawater | MeOH | Brevetoxin | *K. brevis* |
| --- | --- | --- | --- | --- |
| **Ambient** | - | 1.000 | 0.470 | *0.048 |
| **MeOH** | - | - | 0.353 | 0.069 |
| **Brevetoxin** | - | - | - | 1.000 |
| **K. brevis** | - | - | - | - |

C)

| Larvae Settlement | Ambient Seawater | MeOH | Brevetoxin | *K. brevis* |
| --- | --- | --- | --- | --- |
| **Ambient** | - | 0.438 | 0.329 | *0.016 |
| **MeOH** | - | - | 0.997 | 0.383 |
| **Brevetoxin** | - | - | - | 0.499 |
| **K. brevis** | - | - | - | - |

D)

| Larvae Survival | Ambient Seawater | MeOH | Brevetoxin | *K. brevis* |
| --- | --- | --- | --- | --- |
| **Ambient** | - | 1.000 | 0.088 | *0.013 |
| **MeOH** | - | - | 0.079 | *0.012 |
| **Brevetoxin** | - | - | - | 1.000 |
| **K. brevis** | - | - | - | - |
