# Supplementary material for: Exposure to the Florida red tide dinoflagellate, Karenia brevis, and its associated brevetoxins induces ecophysiological and proteomic alterations in Porites astreoides
Source: PLoS One. 2020 Feb 7;15(2):e0228414. doi: 10.1371/journal.pone.0228414 (PMC7006924; doi:10.1371/journal.pone.0228414)
Supplement: S2 Table — (DOCX) [file pone.0228414.s002.docx]

Supplemental Table 2. Database information for publicly available transcriptomes of *Porites* species and constructed database

| Database | # of contig | Min. length | Median length | Max. length | # of hits | Reference |
| --- | --- | --- | --- | --- | --- | --- |
| *Porites australiensis* host and symbiont | 463,214 | 32 | 53 | 14,601 | 832 | Shinzato et al. (2014) |
| *Porites astreoides* heat stress | 80,604 | 32 | 51 | 2,398 | 847 | Kenkel et al. (2013) |
| *Porites astreoides* life history stages | 932,437 | 32 | 51 | 15,904 | 622 | Mansour et al. (2016) |
| *Porites* concatenated nonredundant | 837,531 | 32 | 54 | 1,692 | 1,371 | This study |
