# Supplementary figures and images for: Exposure to the Florida red tide dinoflagellate, Karenia brevis, and its associated brevetoxins induces ecophysiological and proteomic alterations in Porites astreoides
Source: PLoS One. 2020 Feb 7;15(2):e0228414. doi: 10.1371/journal.pone.0228414 (PMC7006924; doi:10.1371/journal.pone.0228414)

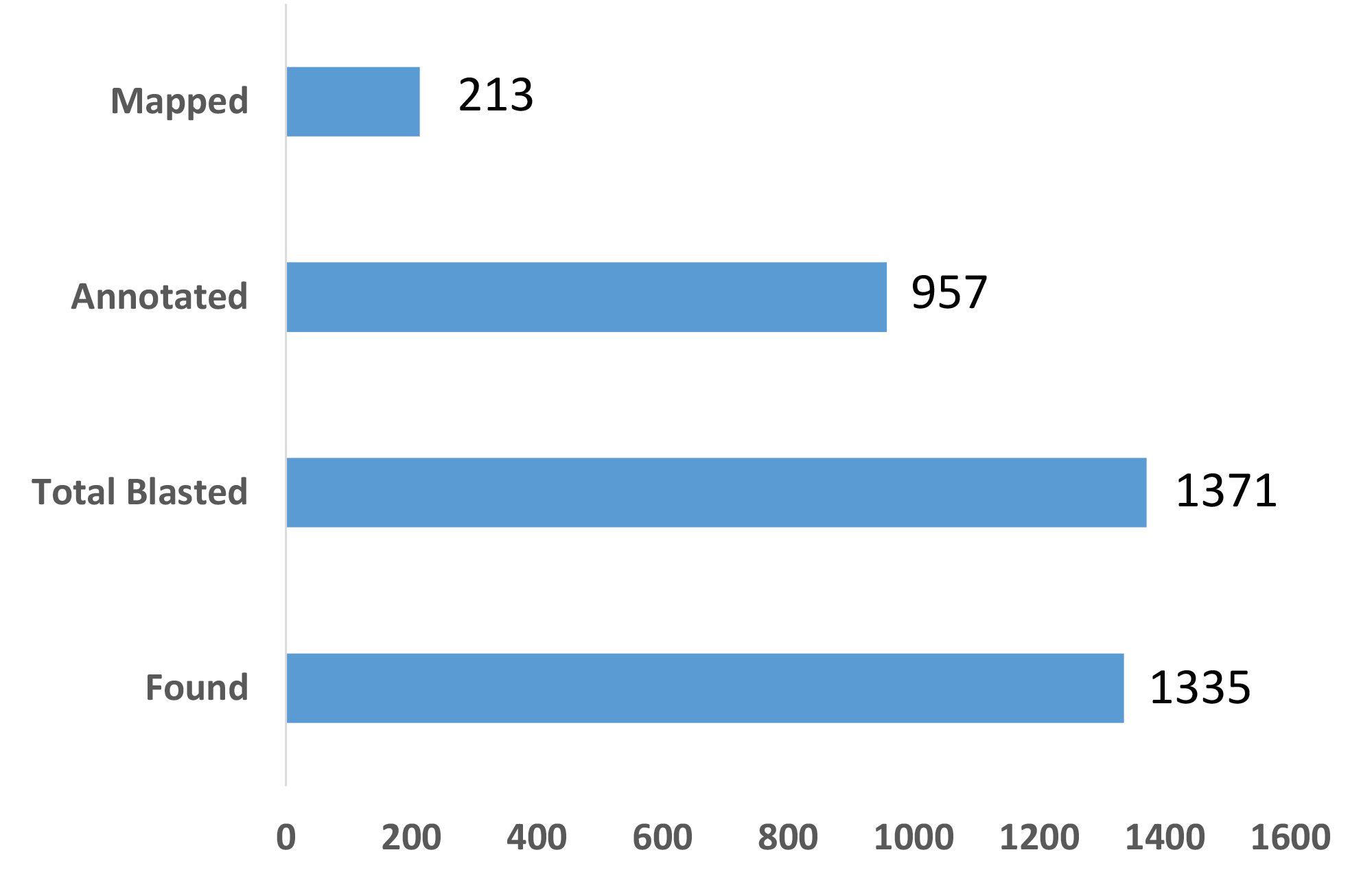

Supplement: S1 Fig — (TIF) [file pone.0228414.s004.tif]

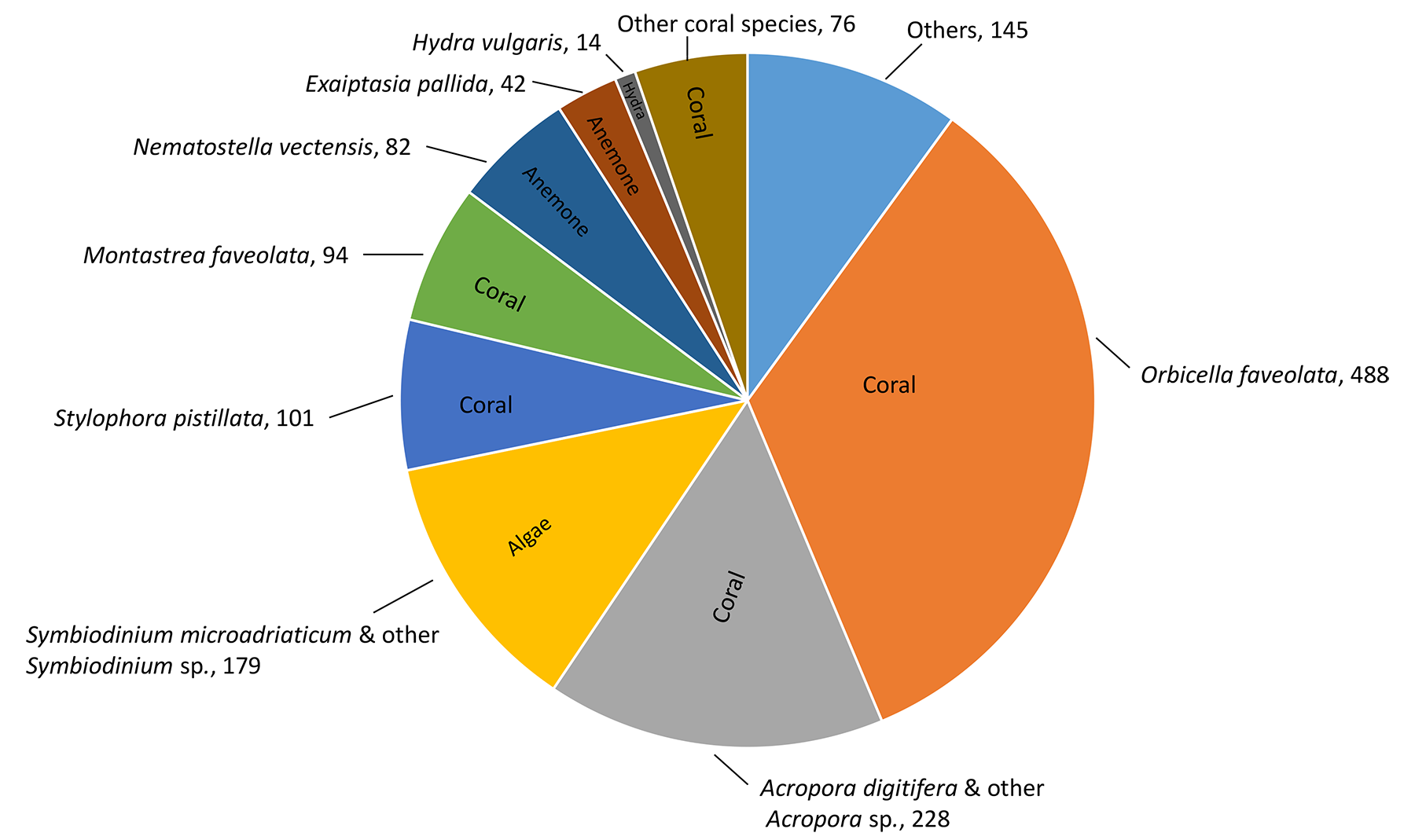

Supplement: S2 Fig — A total of 1,371 protein sequences were identified using the constructed database and were blasted against the NCBI nonredundant protein sequence (nr_v5) database. The majority of hits showed homology to corals including Orbicella faveolata and Acropora sp. (TIF) [file pone.0228414.s005.tif]
